# Cases of Left Against Medical Advice from the Emergency Department of a Tertiary Care Hospital in Kathmandu: A Descriptive Cross-Sectional Study

**DOI:** 10.31729/jnma.5411

**Published:** 2020-12-31

**Authors:** Manish Nath Pant, Saswat Kumar Jha, Sauravi Shrestha

**Affiliations:** 1 Departent of General Practice and Emergency Medicine, Kathmandu Medical College and Teaching Hospital, Kathmandu, Nepal; 2 Jyoti Hospital, Kalimati, Kathmandu, Nepal; 3 Nepal Korea Friendship Municipality Hospital, Madhyapur Thimi, Nepal

**Keywords:** *emergency service*, *informed consent*, *liability*

## Abstract

**Introduction:**

Left against medical advice is a worldwide phenomenon. Patients leaving against Left against medical advice does not provide the health professionals from legal impunity. A well-informed consent should be present with surety that they are well understood by the patient before they leave. The study was undertaken to study the prevalence of patients that leave against medical advice in a tertiary care center.

**Methods:**

This is a descriptive cross-sectional study done in the emergency department of a tertiary care hospital from 1^st^ February 2020 to 31^st^ July 2020. Ethical approval was taken from the Institutional Review Committee (ref. no. 130120205). The sample size was calculated and the convenient sampling method was used. Data were analyzed in the Statistical Package of the Social Sciences version 22. Point estimate at 95% Confidence Interval was calculated along with frequency and proportion for binary data.

**Results:**

Out of 5834 visits, 332 (5.96%) (4.70-7.22 at 95% Confidence Interval) patients left against medical advice. The mean age was 36.48 years (3 days-91 years) and males 173 (52.3%) were prone to leave than females. Only 50 (15.1%) cases had well-informed consent with complications documented. Hundred (30.5%) patients had wanted to come on follow up the next day in the out-patient department while 41 (12.4%) had to leave because of financial reasons. Only seven (2.9%) of well-oriented patients gave their consent and the remaining 233 (97.1%) was by the kin present. Only 76 (23%) patients were sent home with a well-documented medicine prescription.

**Conclusions:**

The proportion of patients who left against medical advice was more than the studies done in the similar setting.

## INTRODUCTION

Leave against medical advice (LAMA) is used for the patients that leave the hospital against the doctor's advice to stay in the hospital and get treated.^1,2^ The person who has opted to go on LAMA should be fully aware of the implications and complications that may occur if treatment does not prevail on time.^3,4^

LAMA is a universal well-recognized problem seen in the inpatients and emergency department which increases mortality and risk of readmissions.^3,4^ At present, there aren't studies on LAMA discharges in Nepal, however, various studies done worldwide have shown the rate of LAMA discharges between 4% to 15% in critically ill patients.^5^

This study aims to study and evaluate the documents of patients that leave on LAMA; if a well-informed consent was written with proper explanation of complications; and if the doctors have sent the patients home with medications to continue their treatment.

## METHODS

This is a cross-sectional study conducted in the emergency room of Kathmandu Medical College and Teaching Hospital from 1^st^ February 2020 to 31^st^ July 2020. Ethical approval was taken from the institutional review committee that was approved in January 2020. All the cases that had gone home against medical advice after signing the preprinted LAMA form was included in the study. The convenience sampling technique was used. The sample size was calculated using the formula:

$$\begin{array}{ll} \text{n} & \text{= }{\text{Z}}^{\text{2}}\times \text{p}\times (\text{1}-\text{p)}/{\text{e}}^{\text{2}}\\ & \text{= }{\left(1.96\right)}^{\text{2}}\times (0.5)\times (1-0.5)/{(\text{0.02\%)}}^{\text{2}}\\ & \text{= }2401 \end{array}$$

Where,

n = sample size
Z = 1.96 at 95% Confidence Interval (CI)
p = population proportion, 50%
e = margin of error, 1%

The total sample size was calculated to be 2401. Since convenience sampling was done to enroll patients, the sample size was doubled to 4802. Adding a 10% non-response rate, the minimum required sample size was 5284. However, 5834 patients were taken in the study.

All the documents of patients that had gone on LAMA during the time frame were collected during the end of the month so that the documents are not missed as at the end of the month all the documents were collected and filed.

If there was missing data for more than two variables, then it was removed from the study. If there were any missing values for a variable, they were replaced with mean/median if the mean or median was less of the values for the variable was less than 5%. Of 332 LAMA cases, 331 were evaluated for patients' characteristics, reasons for LAMA, informed consent documentation for complications of the disease, and prescriptions of medications before sending the patient on LAMA and the department disposing of the patient.

The data were analyzed using the Statistical Package of the Social Sciences version 25. Descriptive statistics were calculated and expressed as frequency and proportion.

## RESULTS

During the period of six months, there were 5834 patients were seen in the ER, among these 332 (5.96%) (4.70-7.22 at 95% Confidence Interval) patients had left on LAMA. The mean age of the patients that went on LAMA was 36.48 years (3 days-92years). Likewise, most of the patients that had gone on LAMA were between 15-29; 97 (29.3%) and 30-44; 98 (29.6%) years of age respectively. Males 173 (52.3%) were seen to have gone on LAMA more frequently than their counterparts. Most of the patients were found to be going on LAMA during the night shift 198 (59.8%) which is from 8 pm to 8 am followed by 100 (30.2%) during the evening shift. The mean duration from entry to LAMA was 4.07 (0.5-16.75) hours (Table 1).

**Table 1:** Patient characteristics (n=331).

Characteristics	Frequency n (%)	Characteristics	Frequency n (%)
Age group		**Duration from entry to LAMA in hours**	4.07 (0.5-16.75)
0-14	40 (12.1)	0-2	70 (21.15)
15-29	97 (29.3)	2-4	126 (38.07)
30-44	98 (29.6)	4-6	84 (25.38)
45-59	42 (12.7)	6-8	31 (9.37)
60-74	28 (8.5)	8-10	8 (2.42)
>75	26 (7.9)	>10	12 (3.63)
**Sex**		Shift of duty	
Male	173 (52.3)	Morning (8am-2pm)	33 (10)
Female	158 (47.7)	Evening (2pm-8pm)	100 (30.2)
		Night (8pm-8am)	198 (59.8)

Hundred and one (30.5%) LAMA patients had been disposed of from the Medicine department followed by Neurosurgery 40 (12.1%), Emergency Medicine 40 (12.1%), Surgery 39 (11.8%), and Psychiatry 33 (10%). Four (1.2%) from ENT and 1 (0.3%) from dental made these departments the least with patients sent on LAMA. Of all the LAMA patients that were sent, only 50 (15.1%) had written documentation of complications of the disease, and only 76 (23%) were sent with prescription of medications. Likewise, from all the cases sent on LAMA by respective departments, dental 1 (100%), obstetric 3 (75%), psychiatry 22 (66.7%), and gynecology 11 (50%) had sent their patients with medicine prescription to continue their treatment at home. The obstetrics department had 3 (75%)and psychiatry department had 18 (45.5%) of all the cases they sent had proper informed consent taken compared to Neurosurgery who had only two (5%) of all their cases sent on LAMA (Table 2).

**Table 2:** Department wise distribution of total LAMA case, informed written complications of the disease and written medicine prescription done.

	Well written complication			Medicine Prescribed	
Department	Yes n (%)	No n (%)	Total	Yes n (%)	No n (%)
**Emergency**	1 (2.5)	39 (97.5)	40	8 (20)	32 (80)
**Medicine**	12 (11.9)	89 (88.1)	101	11 (10.9)	90 (89.1)
**Surgery**	7 (17.9)	32 (82.1)	39	4 (10.3)	35 (89.7)
**Gynaecology**	3 (13.6)	19 (86.4)	22	11 (50)	11 (50)
**Paediatrics**	1 (5.9)	16 (94.1)	17	3 (17.6)	14 (82.4)
**Psychiatry**	15 (45.5)	18 (54.5)	33	22 (66.7)	11 (33.3)
**ENT**	1 (25)	3 (75)	4	1 (25)	3 (75)
**Dental**	0 (0)	1 (100)	1	1 (100)	0 (0)
**Orthopaedics**	5 (75)	25 (25)	30	7 (23.3)	23 (76.7)
**Neurosurgery**	2 (5)	38 (95)	40	5 (12.5)	35 (87.5)
**Obstetrics**	3 (75)	1 (25)	4	3 (75)	1 (25)
**Total**	50 (15.1)	281 (84.9)	331	76 (23)	255 (77)

All of the children and those who were either intubated or had low GCS or confused or under influence of alcohol were signed by their kin. However, of the 239 well-oriented adults, only 7 (2.9%) were signed by the patient themselves, other 233 (97.1%) were signed by their nearest kin (Table 3).

**Table 3:** Consenting capability and person giving he consent for LAMA.

	Pediatric group n (%)	Low GCS^*^ n (%)	Confused n (%)	Intubated n (%)	Well-oriented adults n (%)	Under alcohol influence n (%)	Total n (%)
Self	0 (0)	0 (0)	0 (0)	0 (0)	7 (2.9)	0 (0)	7 (2.1)
Nearest kin	40 (100)	10 (100)	24 (100)	1 (100.0)	233 (97.1)	16 (100.0)	324 (97.9)

* GCS: Glasgow Coma Scale

People opted to go on LAMA because 100 (30.2%) had decided to follow up in OPD the next day and 41 (12.4%) had financial reasons to not stay in the hospital. Patients were sent on LAMA by the treating physician because 29 (8.8%) had wanted to continue their treatment in other centers and 9 (2.7%) didn't want to undergo further treatment or investigations. Eighteen (5.4%) patients were sent home because they didn't have anyone to accompany them in the hospital during treatment (Table 4).

**Table 4:** Reasons for Left Against Medical Advice (n=331)

Reasons for LAMA	Frequency n (%)
Not documented	79 (23.9)
Since its not series, no admissions needed	17 (5.1)
Will follow in OPD	100 (30.2)
Does not want investigation or/and treatment	9(2.7)
Does not want to stay in hospital	6 (1.8)
Financial reasons	41 (12.4)
No caretakers for the patient	18 (5.4)
Baby at home	6 (1.8)
Take to anothercenter	29 (8.8)
Home nearby	6 (1.8)
A similar episode in the past	2 (0.6)
others	18 (5.4)

**Figure 1:**
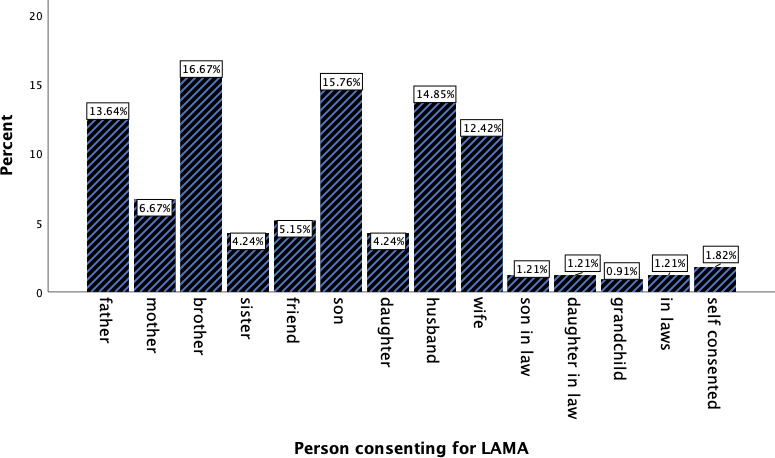
The person who had given consent for the Left Against Medical Advice.

All the cases that were sent on LAMA had their pre-printed declaration of the consent form signed. Only 7 (1.82%) patients had signed the consent form by themselves and all of them were well-oriented adults. The remaining 324 LAMA consent forms were signed by the nearest kin who would be brother 55 (16.6%), son 52 (15.7%), husband 49 (14.8%), father 45 (13.6%), wife 41 (12.4%), mother 22 (6.6%). Similarly, friends 17 (5.1%) were also found to sign the consent for LAMA. Likewise, grandchildren three (0.91%) and in-laws of either male or female 12 (3.6%) had also signed the consent for LAMA (Figure 1).

## DISCUSSION

People leaving against medical advice is not uncommon in Nepal. There is a notion that when the patient goes on LAMA, with documentations completed, the doctor or the health institution is not liable for legal implications even if the patient or the patient party sues which is not true. There have been instances when doctors and hospitals have been sued by patients or their parties even after they have gone on LAMA.^5,6^ This however does not decrease the value of the documents. Thus, the documents should be duly completed and properly signed by the patient or the patient party depending upon the consenting capability of the patient as there is a legal maxim “If you don't write it down, it didn't happen”.^7^

This study is the first of its kind done in a tertiary care center in Nepal to scrutinize the ER documentations of the patients who leave against medical advice. When the documents were analyzed, we looked for written documentation of complications that was explained to the patients and we found only 15.1% had these records, and the remaining 84.9% had only the pre-printed disclosure signed by all going on LAMA. Thus, the majority of the LAMA consent documentation was incomplete in this sense as it cannot demonstrate that the patient/patient's nearest kin has understood the possible risks and courts look for pieces of evidence that the discussion was discussed with the person in question.^7^ This can be related to the fact that many medical professionals do not know, that the failure to obtain and document high-quality informed consent or informed treatment refusals can give rise to a claim of professional negligence.^7^

The code of ethics and professional conduct published in 2017 by NMC states that a mentally sound patient has the right to refuse to consent to treatment, provided that he can exercise his judgment freely and is taken from the patient unless he or she is minor or less than 16 years of age or is mentally unsound or ill to give the decision and this is when the nearest kin who knows the patient as a person who can decide by placing the interest of patients before them.^8^ The refusal should be respected and documented. In this study, there were 72.5% well-oriented adults who could have given their consent, but only 2.9% of all well-oriented adults (240) had signed the consent for themselves, and the remaining 97.1% was signed by the nearest kin available which is comparable to the study from India where the majority 65% of the consented signed for LAMA was by a family member, however, this study lacked to evaluate the consenting capability of the patient for whom the family had consented.^9^

Since it was just the evaluation of documents, we couldn't confirm either the person giving the consent was the nearest person to the patient or the patient had given the authority to decide on his behalf. This is important because the public health service act 2015, in article 2.11.3.a has clearly stated that if only the service recipient is not in a condition to give consent, his or her wife or husband, father or mother, grandfather or grandmother, adult son or daughter, brother or sister so far as available respectively or an available closest person of the service recipient can give the consent. It also has a clause that anyone can give the consent on behalf of the service recipient if he was given the permission or authority to give such consent.^10^

While respecting a patient's right to accept or deny treatment, a doctor has to seek a harm-reduction strategy to ensure the best care possible for their patients. This study was able to demonstrate only 23% patients were sent with medications to continue their treatment. This probably might be due to the misconception of the treating physicians that going on LAMA means denial of treatment as well. The doctors could also have sent the patients with medications on another sheet of paper and just not recorded that in the documents which is also a wrong practice and maybe a source for litigation if something wrong happens to the patient.^7^

The study was able to identify many reasons why people opted for LAMA. The most intriguing reason for the LAMA was that there was no one to take care of the patient at the hospital (5.4%). The hospitals are not willing to take care of the patients because the hospitals and the health professionals don't want to face any threat if any complications occur to the patients in the time of treatment.^11^ This might also be the reason why the doctors aren't convincing enough for admission as sending the patient on LAMA discharge them from their duty to care. Not only that, there is a need fora responsible person to be present in the hospital for payment of the bills as the payment system in Nepal is similar to that in India,^9^ and also for bringing medicines from a pharmacy and take the samples to the lab and bring the reports back to the wards. Most hospitals lack social service units who could have handled all these matters.

The other reasons for them going on LAMA are wanting to follow up in the out-patient department 30.2%, financial reason 12.4%, and wanting to take the patient to another center (8.8%). The financial reason was one of the most common reasons for LAMA in a study in other studies.^9,12^ Even though the government has started mandatory life insurance, it is still not full-fledged and still, the cost of treatment is out of pocket in the private hospitals with limited free care for the poor in the public system, might drive the rate of LAMA from the emergency room abandoning further care.^13,14^ Family concern like having a baby at home was 0.1% in the study from Lebanon compared to our study which was 1.8%.^12^

However, this study is a single centered cross-sectional study in a private medical college, thus cannot be used to generalize the result as we have private hospitals and government-run hospitals as well. This study hadn't expected the COVID-19 pandemic, the willingness to stay in the hospital could be less thus the results could be skewed. The drawback of our emergency was to record the ESI level of the patient as we lack a proper triaging system, we couldn't extract the level of care and severity of the disease the patient had who had gone on LAMA. Since the consenting capacity of the patient was based on GCS and no examination of the consenting capability was done, this cannot be reliable.

## CONCLUSIONS

Overall, LAMA's consent was not fully informed and complete. Thus, to protect oneself from medical litigation, a healthcare professional while respecting the person constitutional right to decide the course of treatment, should ensure the person is mentally sound and understands the severity if not treated and take a full informed consent from the patient or his nearest kin if he/she is not able to give the consent. The disposing department was not sending the patients with a well-written prescription of drugs to alleviate their symptoms. A doctor should be continuing their treatment for the best of their patients even he refuses to continue the treatment at the hospital. There are different modalities of hospital catering treatment to the patients, we need to find out how LAMA cases occur in those centers and how they are being disposed of. Not only that, the reasons for LAMA should also be identified from different centers so that it could be addressed to decrease the morbidity and mortality that arise when patients leave on LAMA.
